# Health technology assessment: the process in Brazil

**DOI:** 10.26633/RPSP.2017.25

**Published:** 2017-03-23

**Authors:** Fernanda Lessa, Marcos Bosi Ferraz

**Affiliations:** 1 Division of Health Economics and Healthcare Management Department of Medicine, Universidade Federal de São Paulo São Paulo Brazil Division of Health Economics and Healthcare Management, Department of Medicine, Universidade Federal de São Paulo, São Paulo, Brazil.

**Keywords:** Technology assessment, biomedical, decision making, organizational, health services administration, health policy, Brazil, Evaluación de la tecnología biomédica, toma de decisiones en la organización, administración de los servicios de salud, política de salud, Brasil, Avaliação da tecnologia biomédica, tomada de decisões gerenciais, administração de serviços de saúde, política de saúde, Brasil

## Abstract

**Objectives.:**

*To describe, analyze, and compare the opinions of decisionmakers involved in the health technology assessment (HTA) process in Brazil in 2011*.

**Methods.:**

*A cross-sectional study was conducted using a structured questionnaire to evaluate the opinions of a convenience sample of health care professionals from both the public and private health care systems (HCS). The survey collected demographic data for each respondent along with their input on national regulations. Data analysis included descriptive statistics, including chi-square tests to compare groups*.

**Results.:**

*Of the 200 completed questionnaires, 65% of the respondents were 31–50 years of age; 36% were HCS managers, 49.3% from the public and 50.7% from the private system. The majority of respondents (85%) considered the time permitted for submission of new technology to be inadequate; 88% also stated that the composition of the evaluation committee needed improvement. Respondents from the private health system more frequently stated that submission times were inappropriate (P = 0.019) and that the deadline for a decision by the committee should be defined (P = 0.021), with a maximum of no more than 180 days / 6 months (P < 0.001)*.

**Conclusions.:**

*Respondents indicated that the HTA process should be improved to meet their expectations. Given that new legislation has been enacted to continuously accept submissions, to make decisions within 180 days, and to expand the committee to represent more stakeholders, most of the respondents concerns have been addressed. This study is valuable as an historical analysis of HTA process improvement. Further surveys are needed to track the new HTA process, its application, and its contribution to health care needs in Brazil*.

## RESUMEN

***Objetivos*.:**

Describir, analizar y comparar las opiniones de los encargados de adoptar las decisiones en el proceso de evaluación de las tecnologías sanitarias llevado adelante en el Brasil durante el 2011.

***Métodos*.:**

Se realizó un estudio transversal en el que se administró un cuestionario estructurado para evaluar las opiniones de una muestra de conveniencia de profesionales de la salud tanto del sistema público como del privado. La encuesta recopiló los datos demográficos de cada encuestado junto con su opinión respecto de los reglamentos nacionales. El análisis de los datos incluyó estadísticas descriptivas, como la prueba de la ji al cuadrado para comparar los datos de los distintos grupos.

**Resultados.:**

De los 200 encuestados que respondieron el cuestionario, el 65 % tenía 31–50 años; el 36 % ocupaba algún cargo gerencial en el sistema de salud, el 49,3 % pertenecía al público y el 50,7 % trabajaba en el sistema privado. La mayoría de los encuestados (85 %) consideraron insuficiente el tiempo permitido para presentar las nuevas tecnologías; el 88 % también declaró que debía mejorar la composición del comité de evaluación. Los encuestados del sistema de salud privado declararon con más frecuencia que los tiempos de presentación habían sido inadecuados (*P* = 0,019) y que era necesario definir un lapso máximo para que el comité tomara su decisión (*P* = 0,021), el que no debía exceder los 180 días o 6 meses (*P* < 0,001).

**Conclusiones.:**

Los encuestados indicaron que es preciso mejorar el proceso de evaluación de las tecnologías sanitarias a fin de satisfacer sus expectativas. En vista de que se han aprobado nuevas leyes que habilitan la presentación continua de nuevas tecnologías, que imponen la toma de decisiones al cabo de 180 días y que amplían el comité para que estén representados más interesados directos, se ha abordado la mayoría de las inquietudes manifestadas por los encuestados. Este estudio es valioso como análisis histórico del mejoramiento de los procesos de evaluación de las nuevas tecnologías. Es preciso emprender más encuestas para llevar a cabo un seguimiento del nuevo proceso, su aplicación y su contribución a las necesidades de atención de salud en el Brasil.

## RESUMO

**Objetivos:**

Descrever, analisar e comparar as opiniões dos tomadores de decisão envolvidos com o processo de Avaliação de Tecnologias em Saúde (ATS) em vigor no Brasil, em 2011.

**Métodos.:**

Estudo transversal conduzido através de questionário estruturado para avaliar as opniões de uma amostra de conveniência composta de profissionais de saúde dos sistemas de saúde público e privado. Foram coletados dados demográficos de todos os respondentes junto com suas opiniões sobre as regulamentações nacionais.

**Resultados.:**

Dos 200 questionários completados, 65% dos respondentes tinham entre 31-50 anos de idade; 36% eram gestores do sistema de saúde: 49.3% do sistema público e 50.7% do privado. A maioria dos respondentes (85%) considerou que o tempo permitido para a submissão de novas tecnologias era inadequado; 88% informou que a composição do comitê de avaliação necessitava melhorias. Respondentes do sistema privado de saúde responderam, com mais frequência, que os tempos de submissão eram inadequados (P=0.019) e que o prazo para a decisão pelo comitê deveria ser definido (P=0.021), com um prazo não superior a 180 dias/ 6 meses (P<0.001).

**Conclusões:**

Respondentes indicaram que o processo de ATS deveria ser melhorado para alcançar suas expectativas. O fato de que uma nova legislação em ATS foi aprovada onde se aceitam submissões de forma contínua, tomada de decisões em até 180 dias, e agregam representantes da sociedade ao comitê de avaliação, demonstra que grande parte das preocupações dos respondentes foi respondida. Este estudo tem seu valor como uma análise histórica da melhoria do processo de ATS no país. Pesquisas adicionais são necessárias para acompanhar o novo processo de ATS, sua aplicação e contribuição às necessidades do sistema de saúde no Brasil.

Health Technology Assessment (HTA) is an investigative process that evaluates the clinical, economic, ethical, and social consequences of using new or existing technologies in health, from research and development to obsolescence. In Brazil, HTA was first mentioned in 1983 during an international seminar that convened representatives from the Pan American Health Organization (PAHO) and the Government of Brazil to discuss the political aspects of HTAs. Topics included the questionable effectiveness of technologies used in health, cost and cost-effectiveness, and the process of technology transfer. Since then, these activities have played a growing role in academia and public policy in Brazil (1–6).

Institutional changes were initiated in 2000 when the Ministry of Health (MoH), the National Agencies for Health Surveillance, and the Regulatory Agency for Private Health Insurance and Plans established the Department of Science and Technology; and then in 2003, when they formed the Secretariat of Science, Technology, and Strategic Inputs. These entities are responsible for policy implementation related to pharmaceuticals and health technology, and are expected to foster industrial and scientific development within Brazil’s Public Health System (SUS). Also in 2003, a Permanent HTA Work Group was established to conduct studies to support and strengthen decision-making related to health technology (7–10).

Later, in 2006, the Commission for the Incorporation of Technologies (the Commission) was created and a process flow was established for incorporating technologies under the auspices of SUS and Supplementary Health System (SHS) (1, 9, 10). In the same year, Brazil took a decisive move into the most important arena for HTA international cooperation when Department of Science and Technology joined the International Network of Agencies in Health Technology Assessment (2, 3).

In 2008, the Secretariat of Science, Technology, and Strategic Inputs was given oversight of the Commission and asked to evaluate and recommend the incorporation, alteration, or exclusion of products for the SUS and SHS procedure lists; to propose the revision of therapeutic guidelines; and to order and carry out HTA-specific studies (6, 11). The process flow for incorporating approved technologies into the health system was also redefined in 2008: proposals could only be submitted during two time-periods annually, i.e., in February–March or in August–September. In turn, the Commission forwarded the process to the Secretariats of the MoH responsible for the preliminary analysis and prepared a technical report with the assistance of the Department of Science and Technology. The report was revised by the Commission, which makes recommendations based on the expected impact of a given technology on the public health system, and a technology’s relevance based on the best scientific evidence. These recommendations are countersigned by the Secretary of the Science, Technology, and Strategic Inputs and forwarded to the Minister of Health for deliberation. After a decision is made, the process returns to the Commission, which sends it to the ministerial secretariat responsible for implementing the measures. When relevant, the Minister of Health sends the recommendation for analysis and a decision by the Regulatory Agency for Private Health Insurance and Plans (6).

Due to advances in HTA, the updating of legislation in the country has had a multi-dynamic character. It is important to mention that this study took place during a period in which both civil society and the MoH agreed that change was needed. A bill was being submitted to Congress that called for alterations to the HTA process, and it ultimately culminated in the enactment of Law 12401/11 (12, 13).

Other HTA studies support the development of evidence-based policies, acknowledging that new ways are needed for linking the technical and political dimensions of health care and for including civil society in the decision to incorporate new technology (6). The MoH has pointed to the creation of a new linkage between scientific policies and public health policy. However, for it to be successful, elected officials representing their populations must participate in the implementation processes, both through the organizations that currently exist and new ones (14).

Initially, this study aimed to analyze the opinions of HTA stakeholders, to identify the uncertainties and challenges of the process, and to understand what strategies were being used to overcome these. However, because this study was conducted just prior to new legislation that implemented significant changes, its purpose is most relevant as an historical analysis of HTA in Brazil.

## MATERIALS AND METHODS

This was a cross-sectional study that used a structured questionnaire to investigate the opinion of a convenience sample of 200 health system decisionmakers in Brazil. The study was carried out in accordance with a protocol approved by the Research Ethics Committee of the Universidade Federal de São Paulo (São Paulo, Brazil). It was a 2-part questionnaire. Part 1 sought to identify the socioeconomic, educational, and demographic profile of the respondents; Part 2 aimed to find collect data on the participants’ opinion of the HTA process in Brazil.

The intended sample for the study was 200 completed questionnaires, a number considered adequate for the analysis described. A database was created with 893 electronic addresses of potential participants, based on the researchers’ professional contacts, university students, and the speakers and participants of events held by the *Grupo Interdepartamental de Economia da Saúde* (Interdepartmental Group on Health Economics), a research unit at the university. Access passwords were created and allocated to potential participants.

Decisionmakers in the health care system were determined according to the following inclusion criteria: professionals who could influence HTA decisions or processes, or whose work could be affected by HTA-related decisions; over 18 years of age; and mentally and physically able to answer the questionnaire. The following exclusion criteria was also applied: those who refused to participate; whose emails addresses invalid; and those with ties to the Interdepartmental Group on Health Economics, e.g., teaching staff, researchers, and/or who assisted with this study.

The participants were divided into nine group established in advance by the researcher according to data on professions available in the databases. These groups were: health care providers; health insurance plan administrators; pharmaceuticals and device industries; regulatory agencies of the MoH and public health departments; commercial diagnostics laboratories and imaging companies; academia; and trade unions and associations of health professionals.

The questionnaire was hosted on an Internet platform. A letter of invitation along with an access password was sent electronically, in a random and balanced sequence, to potential participants among all nine groups. Reminder e-mails were sent every 30 days until a total of 200 completed questionnaires had been submitted.

Descriptive statistics were used to describe the sample, with chi-square tests to study possible associations between variables (*P* = 0.05).

## RESULTS

Invitations for study participation were sent to 597 professionals, 200 of whom responded (response rate of 33.5%). Table 1 describes the demographic characteristics of the participants. Males were 47.0% of the sample. The age group most frequently observed (*n =* 74; 37.0%) was those 41–50 years of age. Most professionals were physicians (*n =* 83; 41.5%), followed by pharmacists (*n =* 33; 16.5%), and nurses (*n =* 20; 10.0%). The majority (*n =* 101; 50.0%) worked in the public health system. In relation to the hierarchical level of their principal function, 20.0% (*n =* 40) were directors and 26.0% (*n =* 52) were managers. Most (*n =* 166; 83.0%) worked in the southeast area of Brazil.

According to Table 1, 60.4% (*n =* 61) of the public sector professionals were women and 54.5% (*n =* 54) of the private sector professionals were men. Of those who worked in the private sector, 90.9% (*n =* 90) were concentrated in the southeast and 51.5% (*n =* 51) were physicians. Of the public health professionals, 12.0% (*n =* 12) worked in the mid-west and 31.7% (*n =* 32) were physicians. In both sectors, most were in the age group of those 41–50 years of age, 34.6% (*n =* 35) in the public sector and 38.4% (*n =* 38) in the private.

**TABLE 1.** Characteristics of 200 health care professionals responding to a questionnaire on the health technology assessment process in Brazil, by type of health care employer, 2010–2011

Characteristics	Health care system					
	Total		Public		Private	
	n	%	n	%	n	%
Gender						
Female	106	53.0	61	60.4	45	45.4
Male	94	47.0	40	39.6	54	54.5
Age group (years)						
41 – 50	73	37.0	35	34.7	38	38.4
31 – 40	56	28.0	28	27.7	28	28.3
51 – 60	37	19.0	20	19.8	17	17.2
< 30	28	14.0	15	14.8	13	13.1
> 60	6	3.0	3	3.0	3	3.0
Geographic area of Brazil						
Southeast	166	83.0	76	75.3	90	90.9
Center-west	15	7.5	12	11.9	3	3.0
South	14	7.0	9	8.9	5	5.1
Northeast	5	2.5	4	3.9	1	1.0
North	0	0.0	0	0.0	0	0.0
Profession						
Physician	83	41.7	32	31.7	51	51.5
Pharmacist	33	16.6	13	12.9	20	20.2
Nurse	20	10.1	13	12.9	7	7.1
Administrator	18	9.0	10	9.9	8	8.1
Engineer	10	5.0	8	7.9	2	2.0
Economist	10	5.0	7	6.9	3	3.0
Other	25	12.6	18	17.8	8	8.1
Main role in health care system						
Manager	72	36.0	26	25.7	46	46.5
Research	31	15.5	24	23.8	7	7.1
Service provider/health care	44	22.0	20	19.8	24	24.2
Technician/Assistant	21	10.5	16	15.8	5	5.1
Teaching	10	5.0	8	7.9	2	2.0
Other	22	11.0	7	6.9	15	15.1

***Source:*** Prepared by the authors from the study data.

As shown in Table 2, most participants were of the opinion that: the HTA review period was inadequate (*n =* 170; 85.0%); the composition of the Commission for HTA review and approval was inadequate (*n =* 176; 88.0%); the entire process should take 6–12 months (*n =* 189; 85.0%); and specific regulations were needed for each type of health technology (*n =* 155; 77.0%).

A comparison of responses by public versus private HCS professionals found a statistically significant association between those in the private services, with regard to: the inadequate submission time period, 92.9% versus 77.2% for those from the public services (*P =* 0.019; X^2^ = 9.967); the evaluation time period as defined by the law, 94.9% versus 85.1% for the public (*P =* 0.021; X^2^ = 5.336); an evaluation period < 6 months, 82.8% versus 58.4% (*P <* 0.001; X^2^ = 14.502); and circumstances under which the MoH should send the recommendation to ANS, 93.9% versus 82.0% (*P <* 0.001; X^2^ = 6.687). Private health system professionals wanted the total analysis time, from registration to recommendation, to be shorter than what those from the public health system were requesting, 54.5% and 37.0% respectively (*P <* 0.001; X^2^ = 9.909). The responses of professionals with ties to government entities and those without such ties were also analyzed (Table 3).

## DISCUSSION

Although Brazil has made important advances in HTA-related policy discussion, formulation, and legislation, there are still many challenges to overcome. The assessment process is still perceived as slow; its methodology is considered incomplete; its scope, insufficient; and its capacity, unable to serve the needs of the both the public and private health systems (4).

Most respondents (85.0%, *n =* 170) considered the allotted timeframe for submitting requests to be inadequate, and there was a statistically significant association between professionals from the private health system and this belief (*P =* 0.019; X^2^ = 9.667). However, since the enactment of Law 12401/11, requests can be submitted at any time; therefore, the feedback captured by this study’s questionnaire has been satisfactorily addressed (12, 13). In addition, the continuous workflow means the process has become more efficient and faster; over time, this could change the perceived “slowness” expressed by stakeholders (4).

Regarding the composition of the Commission, 88.0% of respondents stated it was inadequate for meeting existing demand. There was a statistically significant association between this belief and professionals not directly linked to the government (*P <* 0.001; X^2^ = 9.909). There may be an expectation among this group that they should be consulted and/or participate in the Commission. According to respondents, representatives of professional associations, councils, organizations, and academia should be part of the Commission. This finding concurs with a study stating that government, academia, and specialists are groups that should be included in the HTA process (4).

Law 12401/11 replaced the Commission with the National Commission for the Incorporation of Technology (CONITEC). CONITEC has 13 members, instead of five, including a representative from the National Health Council and another from the Federal Council on Medicine (12, 13, 15). The need for public and patient involvement in the decision-making process is widely recognized given that they stand to either benefit or suffer the consequences of applying a new health technology; furthermore, CONITEC aims to increase transparency and diminish any biases in the process (16). Other countries, such as Australia, Canada, Germany, and the United Kingdom have also made changes that involve the participation of civil society and medical specialists in the HTA process (17–20).

**TABLE 2.** Responses from 200 health care professions who completed a questionnaire on the Health Technology Assessment (HTA) process in Brazil, 2010–2011

	n	%
1. Regarding the time period for submission of new requests		
Inadequate. All submissions should obey the same period and should be done electronically.	92	46.0
Inadequate. All submissions should be done throughout the year.	70	35.0
Adequate	30	15.0
Inadequate. All submissions should obey the same established period.	8	4.0
2. Regarding the composition of the Commission that evaluates submissions		
Inadequate.	176	88.0
Adequate.	24	12.0
3. Regarding the calendar of the Commission that assesses submissions.		
Both the calendar and the agenda should be announced.	158	79.0
Just the calendar should be announced.	26	13.0
Should not be announced.	16	8.0
4. “The times established for analyses should be defined in the legislation.”		
Agree, 6 months.	78	39.0
Agree, 3 months.	63	31.5
Agree, 12 months.	28	14.0
Do not agree.	20	10.0
Agree, 9 months.	11	5.5
5. Referring to the number of votes by which the Commission approves a request.		
Adequate.	82	41.2
Should be considered 80% of valid votes.	64	32.2
Should be by a simple majority.	36	18.1
Should be by consensus.	16	8.5
6. On the approval of the Commission’s recommendations.		
Adequate	101	50.5
Should only be done by the CITECa.	53	26.5
Should only be done by the Secretary of Science, Technology, and Strategic Inputs.	24	12.0
Should only be done by the Minister of Health.	22	11.0
7. “The circumstances under which the Minister of Health submits recommendations for assessment and determination by the ANSb should be clearly defined.”		
Agree.	175	87.5
Disagree.	25	12.5
8. For denying approval of a request: what information should be provided to the applicant?		
Notification and detailed report.	155	77. 9
Notification and justification.	40	20.1
Only a notification.	4	2.0
9. Regarding the appeal process/reconsideration of a decision.		
Inadequate.	181	90.9
It is adequate.	18	9.1
10. How long should the entire process take?		
6 months.	91	45.7
12 months.	78	39.2
18 months.	17	8.5
24 months.	9	4.6
36 months.	4	2.0
11. “Should there be specific regulations for each type of health technology?”		
Yes.	155	77.5
No.	45	22.5
12. Are you aware of the HTA and the incorporation process in Brazil?		
Yes.	78	39.0
Yes, but vaguely.	60	30.0
Yes, extensively.	45	22.5
No.	17	8.5

***Source:*** Prepared by the authors from the study data.

a *Comissão de Incorporação de Tecnologias do Ministério da Saúde* (Commission for Incorporating Technology, Ministry of Health).

b *Agência Nacional de Saúde Suplementar* (National Regulatory Agency for Private Health Insurance and Plans).

Regarding the time allowed to evaluate requests, the legislation in place at the time of this study stated that the Commission should meet pre-established deadlines; however, the terms of deadlines were not specified. Given this, 90.0% (*n =* 180) of participants indicated that deadlines should be clearly defined and stated specifically by the legislation. There was a statistically significant association between professionals in the private system and this statement (*P =* 0.021; X^2^ = 5.336). When asked what the ideal time period should be, professionals in the public system (*P* < 0.001; X^2^ = 14.502) and those directly linked with a government body (*P* = 0.032; X^2^ = 6.874) suggested a longer time period than those in the private system proposed; note that this association was statistically significant.

Possibly, the private sector, which is most often making an HTA request, prefers shorter evaluation periods because it would benefit from having its products included in SUS and SHS more quickly. On the other hand, the public system, usually receiving the request, has a clearer picture of the real demands of the health system, the volume of requests pending analysis, and the time required to carry out a satisfactory evaluation.

Respondents were urged to suggest an assessment time-period that would ensure that only secure and effective technologies would be permitted to enter Brazil’s health care system. Professionals from the private sector and those not directly linked to the government agreed that once a request was registered, the evaluation should take no more than 6 months (*P <* 0.001; X^2^ = 9.909; *P =* 0.002; X^2^ = 12.247, respectively). Again, those from the private health sector preferred a shorter analysis time, while those in the public health system—charged with performing the analyses—preferred a longer time period.

At the time that the responses were collected, the legislation did not stipulate the assessment time for processes. The new legislation established a period of 180 days, extendable for another 90 days, to analyze processes. This period begins on the date of registration, when all required documents have been submitted, and runs through the day that the response is provided. Therefore, the maximum period set forth by the law and the decree, there is a total of 270 days. Given this, in Brazil the HTA process can take 6 months or less or not more than a maximum of 9 months. Australia, Canada, and the United Kingdom require that analysis to be complete in 11–18 months (4, 21–23).

**TABLE 3.** Associations of responses to a questionnaire on the health technology assessment (HTA) process in Brazil, by public or private health care systems and between groups with and without a professional connection to any government entity, Brazil, 2010–2011

Question	Health care system type		*P* value (Chi-square)	Connection with a government entity		*P* value (Chi-square)
	Public %	Private %		Yes %	No %	
Regarding time period for submission of new requests.						
It is adequate	22.77	7.07	0.019 (9.667)	23.81	8.62	0.0056 (7.664)
It is inadequate	77.23	92.93		76.19	91.38	
Regarding the composition of the Commission that evaluates submissions.						
It is adequate	7.34	3.65	NA	12.85	0.39	NA
It is inadequate	92.66	96.35		87.15	99.61	
“The times established for analyses should be defined in the legislation.”						
Disagree	14.85	5.05	0.021 (5.336)	14.29	6.9	NA
Agree	85.15	94.95		85.71	93.1	
“The times established for analyses should be defined in the legislation.”						
Disagree	14.85	5.05	*P* < 0.001 (14.502)	14.29	6.9	0.032 (6.874)
Agree < 6 months	58.42	82.83		60.71	77.59	
Agree > 6 months	26.73	12.12		25.00	15.52	
Announcement of the calendar and the agenda of the Commission						
Should not be announced	10.89	5.05		13.10	4.31	NA
Only the calendar should be announced	11.88	14.14	NA	10.71	14.66	
Both should be announced	77.23	80.81		76.19	81.06	
On the approval of the Commission’s recommendations						
Adequate	57.43	43.43	NA	58.33	44.83	NA
Should be done by the Secretary or Ministry of Health (MoH)	21.78	24.24		20.24	25.00	
Should be done by the CITEC^a^	20.79	32.32		21.43	30.17	
“The circumstances under which the which lead the MoH submit recommendations for assessment and determination by the ANS^b^ should be clearly defined.”						
Agree	82.00	93.94	P < 0.001 (6.6866)	85.71	89.57	NA
Disagree	18.00	6.06		14.29	10.43	
How long should the entire process should take?						
6 months	37.00	54.55	P < 0.001 (9.909)	32.53	55.17	0.002 (12.2468)
12 months	41.00	37.37		44.58	35.34	
> 12 months	22.00	8.08		22.89	9.48	
“Should there be specific regulations for each type of health technology?”						
Yes	76.24	78.79	NA	77.38	77.59	NA
No	23.76	21.21		22.62	22.41	

***Source:*** Prepared by the authors from the study data.

a *Comissão de Incorporação de Tecnologias do Ministério da Saúde* (Commission for Incorporating Technology, Ministry of Health).

b *Agência Nacional de Saúde Suplementar* (National Regulatory Agency for Private Health Insurance and Plans).

### Limitations.

The research for this study was conducted at a time when civil society and the MoH agreed that change in HTA was needed. It is important to note that the study results reflect the attitudes that precipitated the new legislation, and that the subsequent enactment of both the HTA bill and decree would have altered eight of its 11 items. Regardless, this article has value as an historical analysis of the HTA process in Brazil (12, 13).

In addition, 91.5% of respondents claimed to have some knowledge of the HTA process in Brazil. This is not surprising since the study database was built with information from HTA-related professionals. However, a weakness of this strategy is the fact that the sample subjects, mostly working in the southeast part of the country, might not be representative of the various stakeholders’ views nationwide. Since the demographics were only discovered after the data collection and analysis, it remains unknown whether or not this occurred by chance. A chi-squared test to evaluate the significance of this relationship was not performed.

### Conclusions

It is a known fact that Brazil lacks the number of qualified human resources and the institutional dynamics observed in more developed countries. Both of these challenges can hinder efficient and reliable assessments of health technologies (24). Therefore, the new HTA timeframes need to be monitored to verify that they are sufficiently ample for ensuring that only reputable, safe, efficient, and effective technologies are being offered to society.

One of the most important aspects of the new legislation is its call for public consultations on technologies being assessed. Although public consultation is a way for society to participate in and lend more transparency to the decisionmaking process, it is still just a democratic tool whose success strictly depends on the participants’ qualifications, the thoroughness of their input, and on their understanding health system’s needs. However, this success should be followed over time and maybe probably be revised accordingly.

### Disclaimer.

Authors hold sole responsibility for the views expressed in the manuscript, which may not necessarily reflect the opinion or policy of the *RPSP/PAJPH* and/or PAHO.
